# The effect of aged erythrocytes and erythrocyte-derived microparticles on lymphomatous and healthy B cells

**DOI:** 10.1186/1476-9255-12-S1-P8

**Published:** 2015-04-16

**Authors:** Caitlin Hughes, Mark A Vickers, Robert N Barker, Lindsay S Hall

**Affiliations:** 1 University of Aberdeen, IMS, Foresterhill, Aberdeen, AB25 2ZD; 2 Scottish National Transfusion Service, Foresterhill, Aberdeen, AB25 2ZD, UK

## 

Allogeneic blood transfusions have been associated with an increased risk of B cell Non-Hodgkin lymphoma (NHL), but the underlying mechanisms have not been identified. Stored erythrocytes undergo a number of age-related changes, including the release of bioactive membrane vesicles known as microparticles, which are believed to contribute to the complications of transfusion. The aim was to determine the effects of aged erythrocytes and erythrocyte-derived microparticles on lymphomatous and healthy B cell activity, in order to explain their potential role in NHL pathogenesis. Treatment with erythrocytes, although not microparticles, enhanced the proliferation of NHL-derived B cells *in vitro*, as assessed using a BrdU incorporation assay, and by flow cytometric analyses of cell division. Additionally, proliferation of healthy B cells within a population of peripheral blood mononuclear cells was increased by culture with erythrocytes, an effect that appeared to be independent of interactions with T helper cells. Our findings suggest that aged erythrocytes, rather than microparticles, may be important mediators of B cell reactions to stored transfusion products. In particular, the ability of aged erythrocytes to drive B cell proliferation may represent a novel mechanism by which transfusion of stored blood promotes NHL progression. Further research will be necessary to establish the clinical significance of this finding, and to more clearly define the role of B cells in transfusion-related immunomodulation.

